# Sarcopenia predicts adverse outcomes in an elderly population with coronary artery disease: a systematic review and meta-analysis

**DOI:** 10.1186/s12877-021-02438-w

**Published:** 2021-09-14

**Authors:** Qiqi Xue, Jie Wu, Yan Ren, Jiaan Hu, Ke Yang, Jiumei Cao

**Affiliations:** 1 Department of Geriatrics, Ruijin Hospital, Shanghai Jiaotong University School of Medicine, Shanghai, 200025 China; 2 Department of Vascular & Cardiology, Ruijin Hospital, Shanghai Jiaotong University School of Medicine, Shanghai, 200025 China

**Keywords:** Sarcopenia, Coronary artery disease, Outcome, Elderly, meta-analysis

## Abstract

**Background:**

The development of sarcopenia is attributed to normal aging and factors like type 2 diabetes, obesity, inactivity, reduced testosterone levels, and malnutrition, which are factors of poor prognosis in patients with coronary artery disease (CAD). This study aimed to perform a meta-analysis to assess whether preoperative sarcopenia can be used to predict the outcomes after cardiac surgery in elderly patients with CAD.

**Methods:**

PubMed, Embase, the Cochrane library, and Web of Science were searched for available papers published up to December 2020. The primary outcome was major adverse cardiovascular outcomes (MACE). The secondary outcomes were mortality and heart failure (HF)-related hospitalization. The random-effects model was used. Hazard ratios (HRs) with 95% confidence intervals (95%CIs) were estimated.

**Results:**

Ten studies were included, with 3707 patients followed for 6 months to 4.5 ± 2.3 years. The sarcopenia population had a higher rate of MACE compared to the non-sarcopenia population (HR = 2.27, 95%CI: 1.58–3.27, *P* < 0.001; I^2^ = 60.0%, P_heterogeneity_ = 0.02). The association between sarcopenia and MACE was significant when using the psoas muscle area index (PMI) to define sarcopenia (HR = 2.86, 95%CI: 1.84–4.46, *P* < 0.001; I^2^ = 0%, P_heterogeneity_ = 0.604). Sarcopenia was not associated with higher late mortality (HR = 2.15, 95%CI: 0.89–5.22, *P* = 0.090; I^2^ = 91.0%, P_heterogeneity_ < 0.001), all-cause mortality (HR = 1.35, 95%CI: 0.14–12.84, *P* = 0.792; I^2^ = 90.5%, P_heterogeneity_ = 0.001), and death, HF-related hospitalization (HR = 1.37, 95%CI: 0.59–3.16, *P* = 0.459; I^2^ = 62.0%, P_heterogeneity_ = 0.105). The sensitivity analysis revealed no outlying study in the analysis of the association between sarcopenia and MACE after coronary intervention.

**Conclusion:**

Sarcopenia is associated with poor MACE outcomes in patients with CAD. The results could help determine subpopulations of patients needing special monitoring after CAD surgery. The present study included several kinds of participants; although non-heterogeneity was found, interpretation should be cautious.

**Supplementary Information:**

The online version contains supplementary material available at 10.1186/s12877-021-02438-w.

## Background

Sarcopenia is a progressive, generalized skeletal muscle disorder characterized by low muscle strength, low muscle quantity or quality, and low physical performance [1, 2]. The prevalence of sarcopenia is estimated at 5–13% in patients of > 60 years of age and 11–50% in patients of > 80 years of age [3, 4]. Multiple definitions and criteria of sarcopenia are available, using different cutoff points and leading to a lack of standardization and poor application of these definitions in clinical practice [1, 2]. Still, the diagnosis of sarcopenia, using any definition of sarcopenia, is relatively straightforward since it requires the measurement of a combination of muscle mass, muscle strength, and physical performance, and since all definitions use at least two of these parameters [1, 2]. The disease burden from sarcopenia arises from the fact that it is a relatively common condition associated with short-term and long-term adverse effects. Sarcopenia is associated with higher risks of falls and fractures [5] and is a major risk factor for loss of independence in the elderly [6]. The muscular degeneration observed in sarcopenia might ultimately impair daily life activities and adversely affect major surgery outcomes in terms of complications, morbidity, and mortality [7–12].

The development of sarcopenia is attributed to normal aging, but it has multiple aspects [13]. These aspects include type 2 diabetes, obesity, inactivity, reduced number and size of type II muscle fibers, reduced testosterone levels, malnutrition, reduced growth factor levels, and decreased muscle proteins [13–19]. In addition, any disease or condition that will decrease physical activity will contribute to sarcopenia [20–26]. Some risk factors for sarcopenia (i.e., type 2 diabetes, obesity, and inactivity) are also risk factors for coronary artery disease (CAD) [27]. Furthermore, type 2 diabetes, obesity, inactivity, reduced testosterone levels, malnutrition, and reduced growth factor levels are also factors for poor outcomes after a coronary event or after surgery [28–32]. Sarcopenia is associated with lower physical activity and respiratory muscle strength in patients with CAD [33, 34].

To date, several studies have examined sarcopenia as a prognostic factor in patients with CAD [35–37]. Still, the available studies about the impact of sarcopenia on CAD outcomes yield conflicting results [35–44], with studies suggesting a poorer prognosis of CAD in patients with sarcopenia, while other studies suggest no association or associations no longer significant after adjustment for traditional risk factors of poor prognosis. Hence, the exact contributions of sarcopenia to CAD-related health and outcomes are unknown.

We hypothesized that sarcopenia negatively affects the outcomes of elderly patients with CAD who undergo cardiac surgery. Therefore, this meta-analysis aimed to assess whether preoperative sarcopenia can be used to predict the outcomes after cardiac surgery in elderly patients with CAD. The results could help determine subpopulations of patients needing special monitoring after surgery.

## Methods

### Literature search

This systematic review and meta-analysis was performed according to the Preferred Reporting Items for Systematic Reviews and Meta-Analyses (PRISMA) guidelines [45]. The relevant articles were identified based on the PICO principle [46], followed by screening using the eligibility criteria. PubMed, Embase, the Cochrane library, and Web of Science were searched for available papers published up to December 2020 using the MeSH terms of ‘Coronary artery disease’, ‘Coronary heart disease’, and ‘Sarcopenia’, as well as relevant key words.

### Eligibility criteria

The eligibility criteria were 1) population: CAD/CHD patients > 65 years of age, 2) exposure: sarcopenia, 3) non-exposure: non-sarcopenia, 4) primary outcome: major adverse cardiovascular event (MACE), 5) study type: cohort studies, and 6) language: English. Conference abstracts, editorials, comments to the editor, reviews, meta-analyses, and papers with inaccessible full-text were excluded.

### Definition of MACE

The definition of MACE could vary among studies, but MACE is generally defined as a composite endpoint including nonfatal stroke, nonfatal myocardial infarction, cardiovascular and cerebrovascular death, revascularization, and heart failure in this study [47, 48].

### Data extraction

The data were extracted by two investigators according to a pre-specified protocol. The study characteristics (authors, year of publication, country, study design, sex, sample size, sarcopenia index, cutoff value to define sarcopenia, and follow-up duration) and outcomes (MACE, mortality, and heart failure (HF)-related hospitalization) were extracted. If a study reported hazard ratios (HRs), the adjusted HRs with 95% confidence intervals (CIs) were extracted; otherwise, the crude HRs with 95%CIs were obtained.

### Quality of the evidence

Ten studies were included. The level of evidence of all articles was assessed independently by two authors according to the Newcastle-Ottawa Scale (NOS) criteria for quality assessment of cohort studies [49]. Discrepancies in the assessment were resolved through discussion until a consensus was reached. The details were summarized in Supplementary Table S1.

### Statistical analysis

HRs and corresponding 95%CIs were used to summarize the results. Statistical heterogeneity among studies was calculated using Cochran’s Q-test and the I^2^ index. An I^2^ > 50% and Q-test *P* < 0.10 indicated high heterogeneity. The random-effects model was used to avoid possible heterogeneity among studies. The possible publication bias was not assessed by funnel plots and Egger’s test because the number of studies with MACE as the primary outcome was less than 10, in which case the funnel plots and Egger’s test could yield misleading results [50, 51]. A sensitivity analysis was performed by sequentially excluding each study in turn. If the 95%CI of each analysis still included the HR of the initial whole analysis, the results revealed that no single study was outlying and driving the results by itself. All analyses were performed using STATA SE 14.0 (StataCorp, College Station, Texas, USA).

## Results

### Selection of the studies

**Fig. 1:**
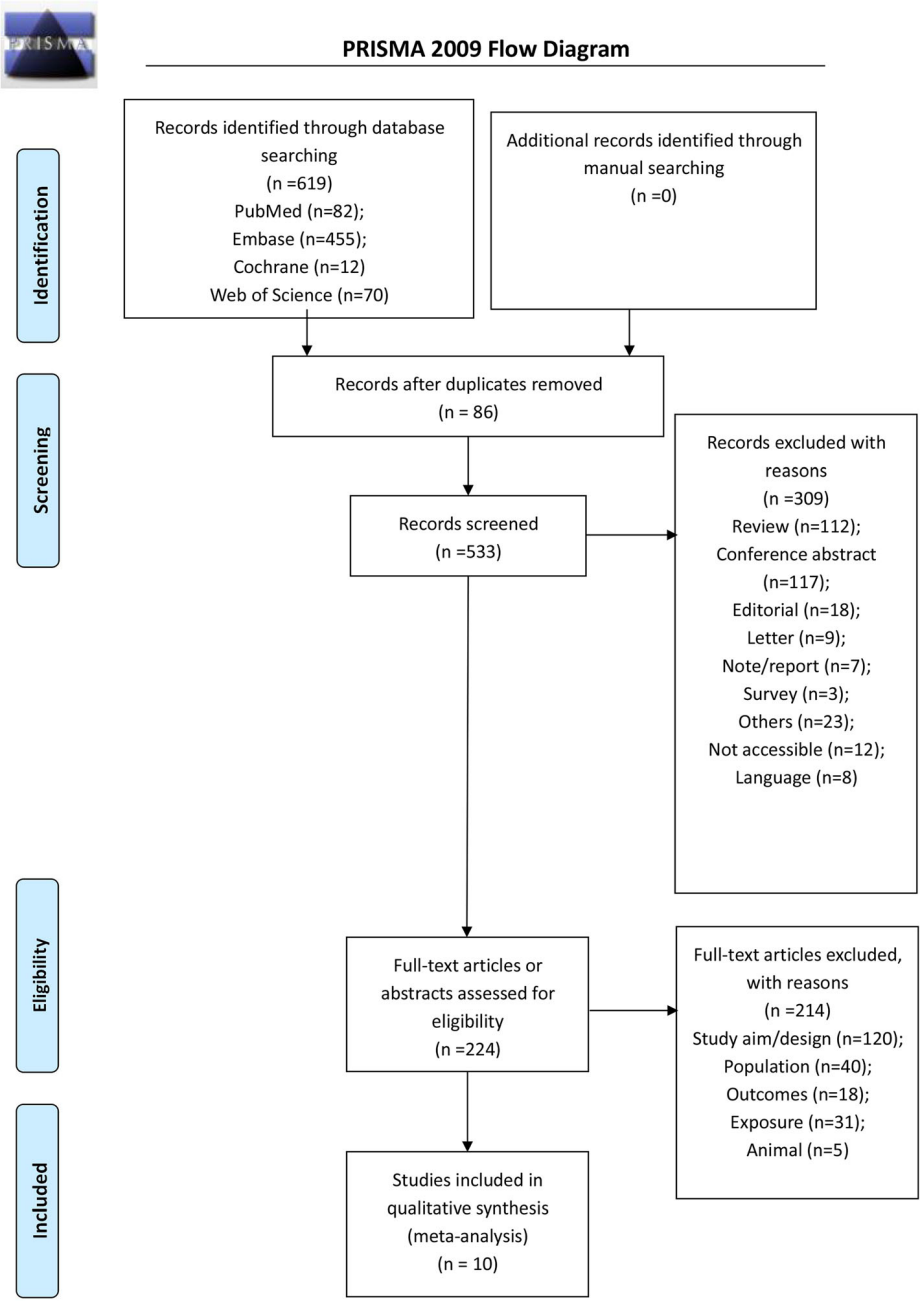
Flow diagram of the study selection process

Figure 1 and Supplementary Table S1 present the study selection process. The initial search yielded 483 records, and 427 records were screened after removing the duplicates. After excluding 250 records, 177 full text articles or abstracts were assessed for eligibility, and 167 were excluded (study aim/design, *n* = 94; population, *n* = 32; outcomes, *n* = 7; exposure, *n* = 31; and animal study, *n =* 3).

Ten studies were included [35, 38–44, 52, 53], with a total of 3707 patients who were followed for 6 months to 4.5 ± 2.3 years (Supplementary Table S2). Nine studies were from Asia [35, 39–44, 52, 53] and one from the United States of America [38]. Supplementary Table S2 shows that the definition of sarcopenia varied among the studies.

Supplementary Table S3 shows that four studies [38–40, 42] scored 8 points on the NOS, and six studies [35, 41, 43, 44, 52, 53] scored 9 points.

### Sarcopenia and MACE after coronary intervention

**Fig. 2:**
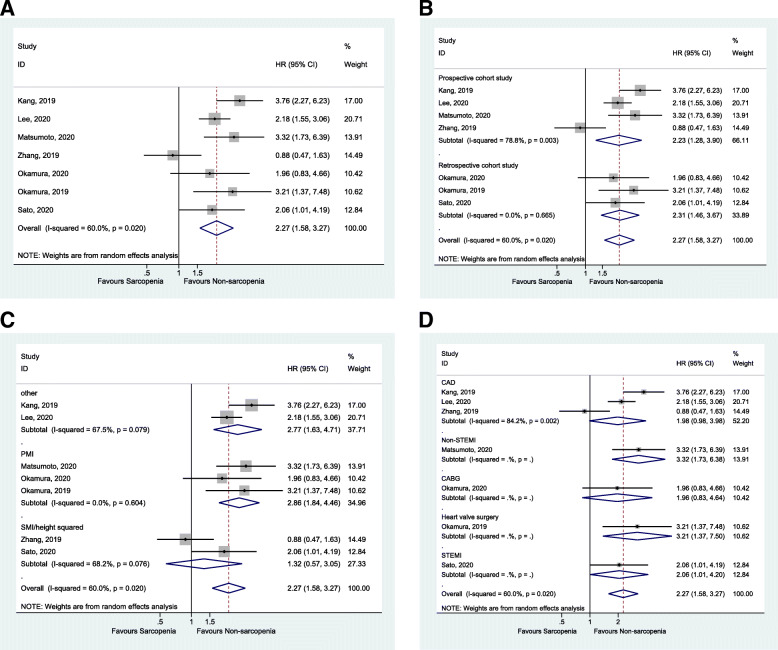
**A**. Forest plot of MACE. **B**. Forest plot of Subgroup analysis of MACE by study type. **C**. Forest plot of subgroup analysis of MACE by sarcopenia index **D**. Forest plot of subgroup analysis of MACE by diagnosis

Seven studies [39–44, 52] analyzed the occurrence of MACE after coronary intervention. The sarcopenia population had a higher rate of MACE compared to the non-sarcopenia population (HR = 2.27, 95%CI: 1.58–3.27, *P* < 0.001; I^2^ = 60.0%, P_heterogeneity_ = 0.02) (Fig. 2A and Table 1). Figure 2B and Table 1 show that this association was observed in prospective cohort studies [41, 43, 44, 52] (HR = 2.23, 95%CI: 1.28–3.90, *P* = 0.005; I^2^ = 78.8%, P_heterogeneity_ = 0.003) and retrospective cohort studies [39, 40, 42] (HR = 2.32, 95%CI: 1.46–3.67, *P* < 0.001; I^2^ = 0%, P_heterogeneity_ = 0.665). When considering the definitions of sarcopenia, the results showed that the association between sarcopenia and MACE was significant when using the psoas muscle area index (PMI) to define sarcopenia [39, 40, 52] (HR = 2.86, 95%CI: 1.84–4.46, *P* < 0.001; I^2^ = 0%, P_heterogeneity_ = 0.604) (Fig. 2C and Table 1), but not when using the skeletal muscle area index (SMI)/height squared [41, 42] (HR = 1.32, 95%CI: 0.57–3.05, *P* = 0.518; I^2^ = 68.2%, P_heterogeneity_ = 0.076) (Fig. 2C and Table 1); the association was also observed when using definitions other than the PMI or SMI/height squared [43, 44] (HR = 2.77, 95%CI: 1.63–4.71, *P* < 0.001; I^2^ = 67.5%, P_heterogeneity_ = 0.079) (Fig. 2C and Table 1).

**Table 1:** Adverse outcomes for sarcopenia versus non-sarcopenia

	N	HR (95%CI)	P	I^2^, %	P_(Heterogeneity)_
**MACE overall**	7	2.273 (1.581–3.268)	< 0.001	60	0.02
Other	2	2.769 (1.630–4.706)	< 0.001	67.5	0.079
PMI	3	2.861 (1.835–4.460)	< 0.001	0	0.604
SMI/height squared	2	1.319 (0.570–3.050)	0.518	68.2	0.076
Prospective cohort	4	2.232 (1.278–3.898)	0.005	78.8	0.003
Retrospective cohort	3	2.315 (1.460–3.670)	< 0.001	0	0.665
CAD	3	1.979 (0.984–3.983)	0.056	84.2	0.002
Non-STEMI	1	3.320 (1.727–6.381)	< 0.001		
CABG	1	1.960 (0.827–4.644)	0.126		
Heart valve surgery	1	3.210 (1.374–7.501)	0.007		
STEMI	1	2.060 (1.011–4.196)	0.046		
**Late mortality**	3	2.152 (0.887–5.223)	0.09	91	< 0.001
Heart valve surgery	2	1.548 (0.7–3.422)	0.281	85.2	0.009
CABG	1	4.250 (2.181–8.283)	< 0.001		
**Death, HF-related hospitalization**	2	1.370 (0.594–3.164)	0.459	62	0.105
**All-cause mortality**	2	1.354 (0.143–12.842)	0.792	90.5	0.001

*HR* hazard ratio, *CI* confidence interval, *MACE* major adverse cardiovascular event, *PMI* psoas muscle area index, *SMI* skeletal muscle area index, *CAD* coronary artery disease, *STEMI* ST-elevated myocardial infarction, *CABG* coronary artery bypass graft, *HF* heart failure

### Sarcopenia, mortality, and HF-related hospitalization after coronary intervention

**Fig. 3:**
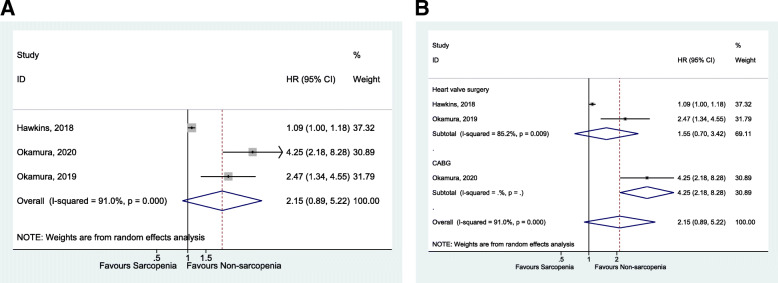
**A**. Forest plot of late mortality. **B**. Forest plot of subgroup analysis of late mortality by diagnosis

**Fig. 4:**
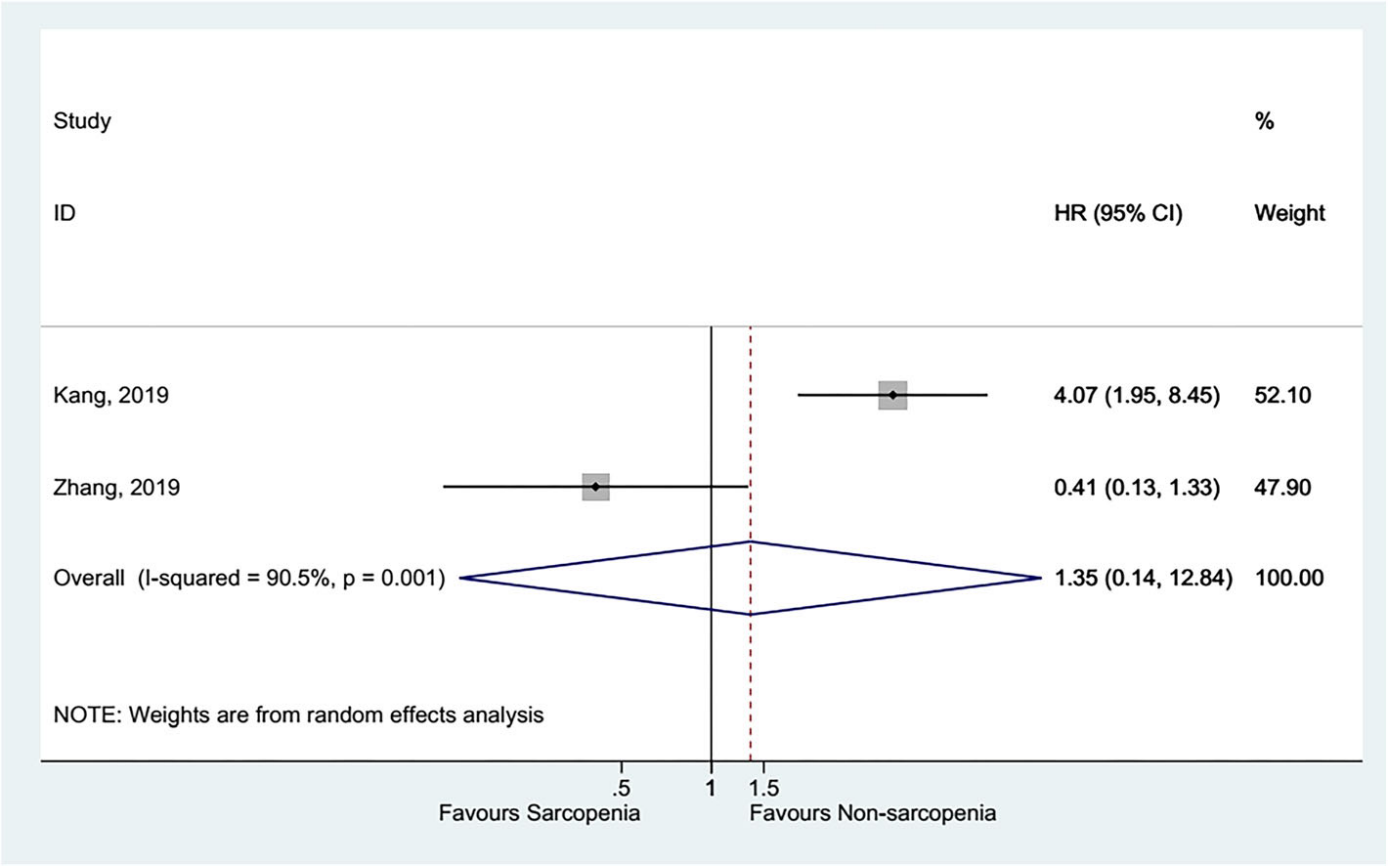
Forest plot of All-cause mortality

**Fig. 5:**
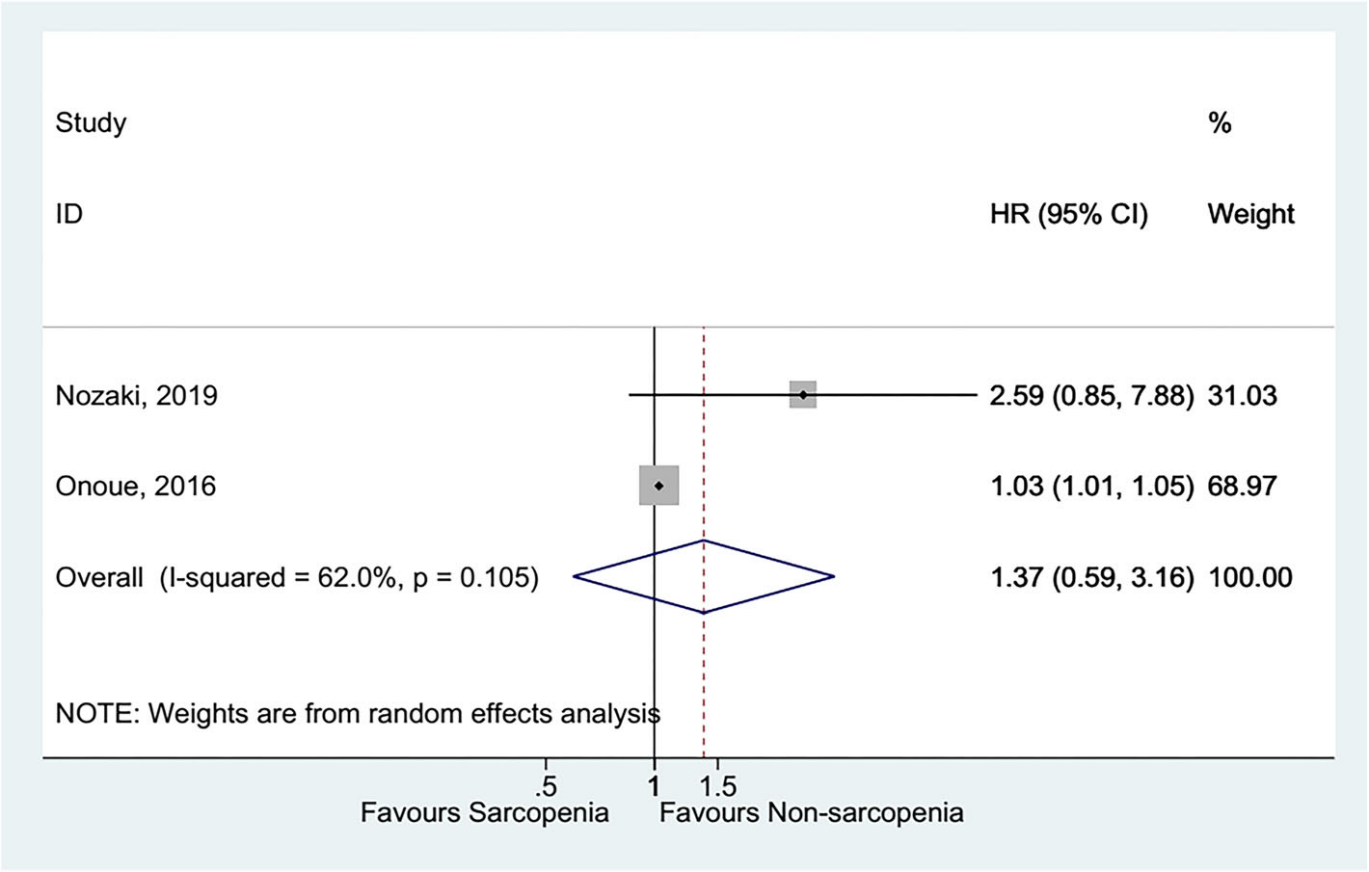
Forest plot of Death, HF-related hospitalization

Sarcopenia was not associated with higher late mortality [38–40] (HR = 2.15, 95%CI: 0.89–5.22, *P* = 0.090; I^2^ = 91.0%, P_heterogeneity_ < 0.001) (Fig. 3 and Table 1), all-cause mortality [41, 43] (HR = 1.35, 95%CI: 0.14–12.84, *P* = 0.792; I^2^ = 90.5%, P_heterogeneity_ = 0.001) (Fig. 4 and Table 1), and death, HF-related hospitalization [35, 53] (HR = 1.37, 95%CI: 0.59–3.16, *P* = 0.459; I^2^ = 62.0%, P_heterogeneity_ = 0.105) (Fig. 5 and Table 1).

### Sensitivity analysis

**Fig. 6:**
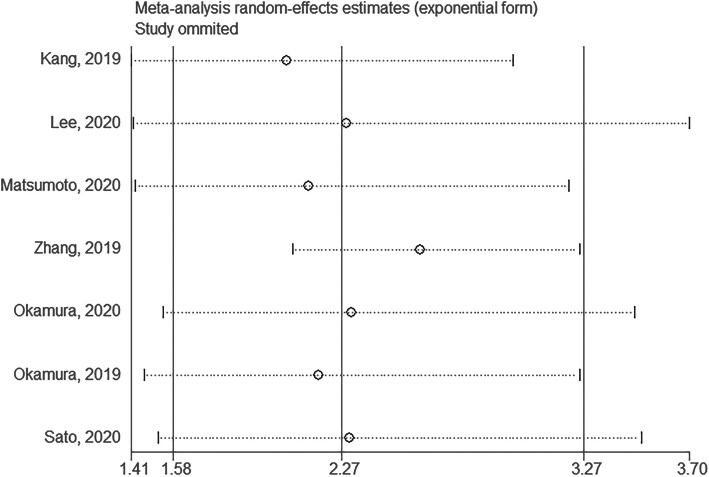
Sensitivity analysis of MACE

The sensitivity analysis suggested that there was no outlying study in the analysis of the association between sarcopenia and MACE after coronary intervention (Fig. 6).

## Discussion

The available studies about the impact of sarcopenia on the outcomes of CAD yield conflicting results. Hence, this meta-analysis aimed to assess whether preoperative sarcopenia can be used to predict the outcomes after cardiac surgery in elderly patients with CAD. The results showed that sarcopenia is associated with poor MACE outcomes in patients with CAD.

These findings highlight the importance of performing a routine physical assessment for risk stratification and sarcopenia in CAD patients. In this context, sarcopenia is a functional status to be detected early in clinical practice, and the importance of PMI is indispensable for identifying simple methods. This is important since a recent meta-analysis showed that sarcopenia could be observed in 31.4% of patients with CAD [54]. Sarcopenia has also been associated with hypertension in older adults [55], with type 2 diabetes [26, 56, 57], and obesity [58, 59]. Hypertension, diabetes, and obesity are also risk factors for the occurrence of CAD [27, 60–62] but are also well-known factors for adverse outcomes in patients with CAD [62–65]. In addition, other factors associated with the development of sarcopenia are also risk factors for poor outcomes after surgery. Indeed, low testosterone levels are associated with increased mortality after CAD and after surgery for CAD [66, 67]. Malnutrition is a factor of poor prognosis in hospitalized patients [68, 69]. Low levels of growth factors are also related to poor outcomes after CAD and surgery [70, 71].

Still, this study showed that only the MACEs after coronary intervention for CAD were affected by sarcopenia, while mortality was not associated. Previous meta-analyses showed that sarcopenia is a risk factor for mortality in the general elderly population [72–74] and a factor of poor prognosis in cancer patients [75, 76]. No previous meta-analysis examined specifically the impact of sarcopenia on MACEs and mortality after coronary intervention. Still, a previous meta-analysis showed that handgrip strength was associated with CAD in the community [77], but a prospective study showed no such association [78]. Still, an association with an increased risk of MACE, even in the absence of increased mortality, signifies a higher disease burden and lower quality of life for the patients [79, 80] and higher healthcare costs for the patients, their family, and society [81].

This meta-analysis revealed wide differences among studies (and even within a single country) regarding the definition of sarcopenia, and these definitions affected the association of sarcopenia with the primary outcome (MACEs). Liu et al. [72] also showed that the method for determining sarcopenia, dual X-ray absorptiometry vs. bioelectrical impedance analysis in their case, affected their results of the association between sarcopenia and all-cause mortality. A recent meta-analysis highlighted the need for the proper diagnosis of sarcopenia and that properly diagnosed sarcopenia was associated with the outcomes after major gastrointestinal surgery [82].

The conclusions of this meta-analysis must be considered along with its limitations. Indeed, meta-analyses inherit all the included studies’ limitations, and some caveats should be considered while interpreting the findings. Because of non-randomized registry data’s intrinsic limitations, the differences in baseline characteristics between groups can affect the outcome. To minimize such biases, two studies [40, 43] performed propensity score-matched analyses, and others used a multivariable logistic regression model. Second, the included sample of 3707 patients was relatively large. Finally, studies that used other and PMI to define sarcopenia demonstrated that the sarcopenia population has a higher MACE rate compared with the non-sarcopenia population. Still, studies performed using SMI/height squared index did not observe the statistical difference between the two groups. Nevertheless, the sensitivity analysis demonstrated no outlying study. Future studies should look to reconcile these definitions of sarcopenia.

A major limitation is that suitable cutoff values for sarcopenia potentially differ among races, sexes, and age groups. In addition, there are many different cutoff values used for the definition of sarcopenia in the literature. Thus, it is difficult to obtain a universal definition of sarcopenia. The cutoff value for sarcopenia was defined as the lowest quartile in most studies [38, 40, 42, 44]. In contrast, some of the other studies performed a receiver operating characteristic (ROC) curve analysis to obtain the optimal cutoff value to define sarcopenia [43, 52]. Because of heterogeneity, a sensitivity analysis was performed and demonstrated no outlying study. In addition, the present study included several kinds of participants such as CAD, NSTEMI/STMI, HF, off-pump CABG, and heart valve surgery. Although non-heterogeneity was found, interpretation should be cautious.

## Conclusion

In conclusion, sarcopenia is associated with poor MACE outcomes in patients with CAD. Still, the definitions of sarcopenia were different among the included studies. Future studies should look to standardize the definition of sarcopenia to achieve better estimations of the associations of sarcopenia with adverse outcomes. Although non-heterogeneity was found, interpretation should be cautious because various types of patients were included.

## Supplementary Information

**Additional file 1: Supplementary Table S1**. Search terms and strategy.

**Additional file 2: Supplementary Table S2.** Literature search and study characteristic.

**Additional file 3: Supplementary Appendix S3**. NOS criteria for quality of cohort studies.

## Data Availability

All data generated or analyzed during this study are included in this published article and its supplementary information files.

## Abbreviations

CAD: Coronary artery disease
CIs: Confidence intervals
HF: Heart failure
HRs: Hazard ratios
MACE: Major adverse cardiovascular event
NOS: Newcastle-Ottawa Scale
PRISMA: Preferred Reporting Items for Systematic Reviews and Meta-Analyses
ROC: Receiver operating characteristic
SMI: Skeletal muscle area index
