# Supplementary material for: Sarcopenia predicts adverse outcomes in an elderly population with coronary artery disease: a systematic review and meta-analysis
Source: BMC Geriatr. 2021 Sep 14;21:493. doi: 10.1186/s12877-021-02438-w (PMC8439080; doi:10.1186/s12877-021-02438-w)
Supplement: Supplementary file 1 — Additional file 1: Supplementary Table S1. Search terms and strategy. [file 12877_2021_2438_MOESM1_ESM.docx]

**Supplementary Table S1. Search terms and strategy**

This appendix provides the questions searched for, an overview of the search strategy, and detailed search terms and logic used in each database (PubMed, Embase, Cochrane and Web of Science).

|  |  | **Search strategy** | **Numbers** |
| --- | --- | --- | --- |
| **PubMed** |  |  |  |
| **Patient** | **#1** | Artery Disease, Coronary OR Artery Diseases, Coronary OR Coronary Artery Diseases OR Left Main Coronary Artery Disease OR Left Main Disease OR Left Main Diseases OR Left Main Coronary Disease OR Coronary Arteriosclerosis OR Arterioscleroses, Coronary OR Coronary Arterioscleroses OR Atherosclerosis, Coronary OR Atheroscleroses, Coronary OR Coronary Atheroscleroses OR Coronary Atherosclerosis OR Arteriosclerosis, Coronary OR "Coronary Artery Disease"[Mesh] | 216,408 |
|  | **#2** | Coronary Diseases OR Disease, Coronary OR Diseases, Coronary OR Coronary Heart Disease OR Coronary Heart Diseases OR Disease, Coronary Heart OR Diseases, Coronary Heart OR Heart Disease, Coronary OR Heart Diseases, Coronary OR "Coronary Disease"[Mesh] | 356,489 |
|  | **#3** | #1 OR #2 | 371,258 |
| **Exposure** | **#4** | "Sarcopenia"[Mesh] OR Sarcopenia | 12,286 |
| **All** | **#5** | #3 AND #4 | 82 |
| **Embase** |  |  |  |
| **Patient** | **#1** | 'coronary artery disease'/exp OR 'coronary disease' | 369,980 |
|  | **#2** | 'coronary heart disease'/exp OR 'coronary heart disease' | 742,041 |
|  | **#3** | #1 OR #2 | 888,596 |
| **Exposure** | **#4** | 'sarcopenia'/exp OR ‘sarcopenia’ | 20,215 |
| **All** | **#5** | #3 AND #4 | 455 |
| **Cochrane** |  |  |  |
| **Patient** | **#1** | MeSH descriptor: [coronary artery disease] explode all trees | 6,696 |
|  | **#2** | Arteriosclerosis, Coronary OR Coronary Arteriosclerosis OR Coronary Atherosclerosis OR Atherosclerosis, Coronary OR Atherosclerosis, Coronary OR Arteriosclerosis, Coronary OR Coronary Atherosclerosis OR Coronary Arteriosclerosis OR Artery Disease, Coronary OR Diseases, Coronary Artery OR Coronary Artery Diseases OR Artery Diseases, Coronary OR Disease, Coronary Artery | 26,573 |
|  | **#3** | MeSH descriptor: [coronary disease] explode all trees | 14,067 |
|  | **#4** | Heart Diseases, Coronary OR Coronary Diseases OR Disease, Coronary Heart OR Coronary Heart Diseases OR Heart Disease, Coronary OR Diseases, Coronary OR Coronary Heart Disease OR Disease, Coronary OR Diseases, Coronary Heart | 38,433 |
| **Exposure** | **#5** | MeSH descriptor: [Sarcopenia] explode all trees | 515 |
|  | **#6** | Sarcopenia or Sarcopenias | 1,700 |
|  | **#7** | #1 OR #2 OR #3 OR #4 | 40,093 |
|  | **#8** | #5 OR #6 | 1,700 |
| **All** | **#9** | #7 AND #8 | 12(11 trials) |
|  |  |  |  |

**Web of Science**

| # 5 | **[70](http://apps.webofknowledge.com/summary.do?product=WOS&doc=1&qid=112&SID=5EI5uqqxUkVN8TbC3td&search_mode=AdvancedSearch&update_back2search_link_param=yes)** | #1 AND #4  *索引=SCI-EXPANDED, SSCI 时间跨度=所有年份* | [编辑](http://apps.webofknowledge.com/WOS_AdvancedSearch_input.do?product=WOS&SID=5EI5uqqxUkVN8TbC3td&search_mode=AdvancedSearch&replaceSetId=5&editState=init) |  |  |
| --- | --- | --- | --- | --- | --- |
|  | | | | | |
| # 4 | **[137,778](http://apps.webofknowledge.com/summary.do?product=WOS&doc=1&qid=111&SID=5EI5uqqxUkVN8TbC3td&search_mode=AdvancedSearch&update_back2search_link_param=yes)** | #2 OR #3  *索引=SCI-EXPANDED, SSCI 时间跨度=所有年份* | [编辑](http://apps.webofknowledge.com/WOS_AdvancedSearch_input.do?product=WOS&SID=5EI5uqqxUkVN8TbC3td&search_mode=AdvancedSearch&replaceSetId=4&editState=init) |  |  |
|  | | | | | |
| # 3 | **[127,220](http://apps.webofknowledge.com/summary.do?product=WOS&doc=1&qid=110&SID=5EI5uqqxUkVN8TbC3td&search_mode=AdvancedSearch&update_back2search_link_param=yes)** | AB=(Coronary Diseases OR Disease, Coronary OR Diseases, Coronary OR Coronary Heart Disease OR Coronary Heart Diseases OR Disease, Coronary Heart OR Diseases, Coronary Heart OR Heart Disease, Coronary OR Heart Diseases, Coronary )  *索引=SCI-EXPANDED, SSCI 时间跨度=所有年份* | [编辑](http://apps.webofknowledge.com/WOS_AdvancedSearch_input.do?product=WOS&SID=5EI5uqqxUkVN8TbC3td&search_mode=AdvancedSearch&replaceSetId=3&editState=init) |  |  |
|  | | | | | |
| # 2 | **[96,182](http://apps.webofknowledge.com/summary.do?product=WOS&doc=1&qid=109&SID=5EI5uqqxUkVN8TbC3td&search_mode=AdvancedSearch&update_back2search_link_param=yes)** | AB=(Artery Disease, Coronary OR Artery Diseases, Coronary OR Coronary Artery Diseases OR Left Main Coronary Artery Disease OR Left Main Disease OR Left Main Diseases OR Left Main Coronary Disease OR Coronary Arteriosclerosis OR Arterioscleroses, Coronary OR Coronary Arterioscleroses OR Atherosclerosis, Coronary OR Atheroscleroses, Coronary OR Coronary Atheroscleroses OR Coronary Atherosclerosis OR Arteriosclerosis, Coronary)  *索引=SCI-EXPANDED, SSCI 时间跨度=所有年份* | [编辑](http://apps.webofknowledge.com/WOS_AdvancedSearch_input.do?product=WOS&SID=5EI5uqqxUkVN8TbC3td&search_mode=AdvancedSearch&replaceSetId=2&editState=init) |  |  |
|  | | | | | |
| # 1 | **[14,144](http://apps.webofknowledge.com/summary.do?product=WOS&doc=1&qid=97&SID=5EI5uqqxUkVN8TbC3td&search_mode=AdvancedSearch&update_back2search_link_param=yes)** | TS=(Sarcopenia)  *索引=SCI-EXPANDED, SSCI 时间跨度=所有年份* | [编辑](http://apps.webofknowledge.com/WOS_AdvancedSearch_input.do?product=WOS&SID=5EI5uqqxUkVN8TbC3td&search_mode=AdvancedSearch&replaceSetId=1&editState=init) |  |  |
