# Supplementary material for: Sarcopenia predicts adverse outcomes in an elderly population with coronary artery disease: a systematic review and meta-analysis
Source: BMC Geriatr. 2021 Sep 14;21:493. doi: 10.1186/s12877-021-02438-w (PMC8439080; doi:10.1186/s12877-021-02438-w)
Supplement: Supplementary file 2 — Additional file 2: Supplementary Table S2. Literature search and study characteristic. [file 12877_2021_2438_MOESM2_ESM.docx]

**Supplementary Table S2.** Literature search and study characteristic

| Study | Design | Country | Population | Sample size | | | Age (year, mean or median) | | Gender, male (%) | | Index | Cutoff value | Follow-up | Adjusted variables |
| --- | --- | --- | --- | --- | --- | --- | --- | --- | --- | --- | --- | --- | --- | --- |
|  |  |  |  | Total | Sarcopenia | Non-sarcopenia | Sarcopenia | Non-sarcopenia | Sarcopenia | Non-sarcopenia |  |  |  |  |
| Kang, 2019 | Prospective cohort study | Korea | CAD, post PCI | 475 | 141 | 334 | 70.4 (11) | 64 (9.2) | 100 (70.9) | 230 (68.8) | LI SMI | Men: 31cm2/m2, Women: 25 cm2/m2 | 3 years | Age, sex, body mass index (BMI), presentation as MI, hypertension, diabetes, dyslipidemia, previous malignancy, clopidogrel and statins at discharge, multivessel disease, creatinine clearance <60 mL/min, LVEF <50%, total cholesterol, number of treated lesions and treated vessels, number of implanted stents, average stent diameter, total stent length, and implantation of second-generation drug-eluting stents |
| Lee, 2020 | Prospective cohort study | Korea | CAD, post PCI | 1,086 | 273 | 813 | 73.5 (6.1) | 71.9 (5.1) | 170 (62.3) | 511 (62.9) | Serum creatinine/serum cystatin C ratio | / | 3 years | Age, sex, BMI, diabetes mellitus, hypertension, peripheral vascular disease, previous MI or PCI, current smoking, previous congestive heart failure, clinical diagnosis, diseased vessel extent and total stent length |
| Matsumoto, 2020 | Prospective cohort study | Japan | NSTEMI | 132 | 62 | 70 | / | / | / | / | PMI | 772mm2/m2 | 2.4 (1.1-4) years | BMI (kg/m2), Hyperlipidemia, hemoglobin (g/dL), serum creatinine (mg/dL), serum albumin (g/dL), high-sensitivity CRP (mg/dL), LVEF＜50%. |
| Nozaki, 2019 | Prospective cohort study | Japan | HF | 191 | / | / | 73.3 (7.3) | | 136 (71%) | | SMI/height squared | Men: 7.0 kg/m2, Women: 5.7 kg/m2 | 0.6 years |  |
| Onoue, 2016 | Prospective cohort study | Japan | HF | 119 | 82 | 37 | 77.6 (5.4) | 72 (5.9) | 53 (65) | 20 (54) | Sarcopenia score by Ishii et al. | Men:≥105; Women:≥120 | 1.4 (0.58-1.96) years | Age (years), Sex (male), BMI (kg/m2), NYHA functional class Sarcopenia score Coronary artery disease Hypertension Dyslipidemia, Diabetes mellitus Current smoker Hemoglobin (g/dl) Albumin (g/dl) eGFR (ml/min/1.73 m2) ln (BNP) ln (hs-TnT) ln (hs-CRP) LVEF (%) LVDd (mm) E/e′ |
| Zhang, 2019 | Prospective cohort study | China | CAD | 345 | 78 | 267 | 79.5 (6.7) | 72 (6.7) | 32 (41) | 176 (65.9) | ASMI/height squared | Men: 7.0 kg/m2, Women: 5.7 kg/m2 | 0.96 (0.82-1.08) years | Sex, age, and the Charlson comorbidity index |
| Hawkins, 2018 | Retrospective cohort study | USA | SAVR with or without coronary bypass | 240 | 60 | 180 | 81 (5.9) | 80 (7.4) | 36 (60) | 107 (59.4) | PMI | Men:9.09cm2/m2; Women: 6.96cm2/m2 | / | Major morbidity, prolonged ventilation, length of stay, discharge to a facility and hospital cost. |
| Okamura, 2020 | Retrospective cohort study | Japan | Off-pump CABG | 304 | 76 | 228 | 69.9 (8.9) | 66.6 (9.7) | 63 (82.9) | 190 (83.3) | PMI | Men: 215 mm2/m2, Women: 142 mm2/m2 | 4.5±2.3 years | age, sex, BMI, hypertension, diabetes, diabetes treated with insulin, dyslipidaemia, smoking history <3 months, prior MI, prior percutaneous coronary intervention (PCI), left main coronary artery lesion, 3-vessel disease, recent MI <3 months, unstable angina pectoris, atrial fibrillation, cerebrovascular disease, chronic kidney disease (serum creatinine >1.5 mg/dl), haemodialysis, peripheral artery disease, prior cardiac surgery, left ventricular (LV) end-diastolic dimension, LV end-systolic dimension, LV ejection fraction, serum haemoglobin, serum albumin, use of bilateral IMAs, total arterial revascularization, use of IABP and sarcopenia |
| Okamura, 2019 | Retrospective cohort study | Japan | Heart valve surgery via median sternotomy | 428 | 107 | 321 | 77 (4.6) | 76 (4.3) | 55 (51.4) | 167 (52) | PMI | / | 3.4 years | Age; sex; body surface area; BMI < 18.5 kg/m2, BMI >30.0 kg/m3 |
| Sato, 2020 | Retrospective cohort study | Japan | STEMI | 387 | 97 | 290 | 74 (9) | 63 (13) | 79 (81.4) | 236 (81.4) | ASMI/height squared | Men: ≤ 6.64 kg/m2, Women: ≤ 5.06 kg/m2 | 2.8 (1.0-3.9) years | Age, gender, dyslipidemia, DM, past history of MI, Hb, serum creatinine, hsCRP, peak CK, Killip classification, LVEF, SYNTAX score, prevalence of statin use at discharge, body mass index, body fat percentage |
| Abbreviation: BMI, body mass index; STEMI, ST-segment elevation myocardial infraction; PMI, psoas muscle area index ; SMI, skeletal muscle mass index; L1, first lumbar vertebra; MI, myocardial infarction; HF, heart failure; SAVR, Surgical aortic valve replacement; CABG, coronary artery bypass grafting; NSTEMI, non‒ST-segment‒elevation myocardial infarction; DM: diabetes mellitus, MI: myocardial infarction, Hb: hemoglobin, hsCRP: high-sensitivity C-reactive protein, CK: creatine kinase, LVEF: left ventricular ejection fraction; MACCE, major adverse cardiac and cerebrovascular events; ACEI, angiotensin-converting enzyme inhibitor; ARB, angiotensin receptor blocker; BMI, body mass index; BNP, brain natriuretic peptide; eGFR, estimated glomerular filtration rate; hs-CRP, high-sensitivity C-reactive protein; hs-TnT, high-sensitivity cardiac troponin T; ln, logarithm; LVEF, left ventricular ejection fraction; LVDd, left ventric- ular end-diastolic diameter; NYHA, New York Heart Association; SYNTAX, SYNergy between PCI with TAXUS and Cardiac surgery) | | | | | | | | | | | | | | |

STEMI, ST-segment elevation myocardial infarction; PMI, psoas muscle area index; SMI, skeletal muscle mass index; L1, first lumbar vertebra; MI, myocardial infarction; HF, heart failure; SAVR, surgical aortic valve replacement; CABG, coronary artery bypass grafting; NSTEMI, non-ST-segment-elevation myocardial infarction
