# Supplementary material for: Sarcopenia predicts adverse outcomes in an elderly population with coronary artery disease: a systematic review and meta-analysis
Source: BMC Geriatr. 2021 Sep 14;21:493. doi: 10.1186/s12877-021-02438-w (PMC8439080; doi:10.1186/s12877-021-02438-w)
Supplement: Supplementary file 3 — Additional file 3: Supplementary Appendix S3. NOS criteria for quality of cohort studies. [file 12877_2021_2438_MOESM3_ESM.docx]

**Supplementary Appendix S3**. NOS criteria for quality of cohort studies

| Study | Representativeness of the exposed cohort | Selection of the non-exposed cohort | Ascertainment of exposure | Demonstration that outcome of interest was not present at the start of the study | Comparability of cohorts based on the design or analysis | Assessment of outcome | Was follow-up long enough for outcomes to occur | Adequacy of follow up of cohorts | Total quality scores |
| --- | --- | --- | --- | --- | --- | --- | --- | --- | --- |
| Kang, 2019 [41] | ☆ | ☆ | ☆ | ☆ | ☆☆ | ☆ | ☆ | ☆ | 9 |
| Lee, 2020 [42] | ☆ | ☆ | ☆ | ☆ | ☆☆ | ☆ | ☆ | ☆ | 9 |
| Matsumoto, 2020 [50] | ☆ | ☆ | ☆ | ☆ | ☆☆ | ☆ | ☆ | ☆ | 9 |
| Nozaki, 2019 [51] | ☆ | ☆ | ☆ | ☆ | ☆☆ | ☆ | ☆ | ☆ | 9 |
| Onoue, 2016 [33] | ☆ | ☆ | ☆ | ☆ | ☆☆ | ☆ | ☆ | ☆ | 9 |
| Zhang, 2019 [39] | ☆ | ☆ | ☆ | ☆ | ☆☆ | ☆ | ☆ | ☆ | 9 |
| Hawkins, 2018 [36] | ☆ | ☆ | ☆ | / | ☆☆ | ☆ | ☆ | ☆ | 8 |
| Okamura, 2020 [37] | ☆ | ☆ | ☆ | / | ☆☆ | ☆ | ☆ | ☆ | 8 |
| Okamura, 2019 [38] | ☆ | ☆ | ☆ | / | ☆☆ | ☆ | ☆ | ☆ | 8 |
| Sato, 2020 [40] | ☆ | ☆ | ☆ | / | ☆☆ | ☆ | ☆ | ☆ | 8 |
